# A Case of Chronic Dysentery Terminating in Perforation of the Rectum

**Published:** 1871-11

**Authors:** Isaac W. Angel

**Affiliations:** Charleston, S. C.


### A CASE OF CHRONIC DYSENTERY TERMINATING IN PERFORATION OF THE RECTUM.

BY ISAAC W. ANGEL, M. D., OF CHARLESTON, S. C.

On the first day of September, I was informed of the death of Mary Fevers, a colored woman, 58 years of age, a native and resident of Charleston, who had been under my care for the past six months, at different intervals of time. She was of medium size and average height, apparent weight, about 120 pounds. When first under my care, she described her symptoms as intense pain "in the end of the back bone," and her disease as a continued diarrhœa, "with bearing down pain, doing very little at a time," from which I concluded that it was a case of dysentery, for which I put her on the usual treatment of hydro sub-mur. et opii, which appeared to check the dysenteric stools, but did not relieve the pain. I also advised blisters over the sacrum, to be repeated from time to time, as should be required, cautioning her to abstain from vegetable food, and as the season was advancing to remember not to indulge in melons and fruits. I lost sight of the case for some time, and having heard that she was better, was in hopes that she was permanently relieved. Some time in August she came to my office and complained that the symptoms had returned, especially the "pain in the end of the backbone." I repeated the former treatment, and as she complained of debility, advised her to take a little stimulus in the day, whenever she felt weak. On the first of September I was informed, that early that morning, she complained of colic, feeling cold, vomiting, etc., continued jactitation, and finally collapsed, ending in her death. One point struck me just here, as one of remarkable interest. This patient was a washerwoman, and on the evening previous to her death, was carrying on her usual business of washing, complaining of no more pain than usual. The suddenness of her death, and the fact that she had not indulged in more than her ordinary diet, made me particularly anxious to enquire what was the cause of death, and to demand a post mortem examination, which was readily granted:

POST-MORTEM, SEPT. 1ST, 1871.

The body appeared quite normal, with the exception of great distension of the abdomen, which seemed about the size of that of a pregnant woman, between the fifth and sixth months of pregnancy.

Dr. R. P. Huger, assisting me, an incision was made from the eusiform cartilage to the os pubis; upon passing through the abdominal muscles, a large amount of gas escaped giving forth a very offensive fœcal odor; the colon was immensely distended and without actual measurement, I should say, was about five inches in diameter. Covering the intestines was a large collection of semi-fluid fœcal matter, to which we must attribute the peritonitis that was presented to our view. This presence of fœcal matter within the peritoneal cavity at once directed our search for a perforation as the cause of the same. Commencing at the stomach, I examined each part of the intestinal canal, inch by inch. Our attention was directed to the extreme thinness of the intestines in several places, especially one or two of Peyers glands, which were so thin as to cause us to look upon them as the point of perforation, but continuing still downward so as to leave no point unsearched, we found the pelvic cavity more or less filled with fluid fœcal matter, and the rectum almost black. Passing the finger downward, we discovered a perforation in the right posterior wall of the rectum, large enough to admit the ends of two fingers. Taking the rectum out and examining it, the perforation presented the appearance of a gun-shot wound, as regards its torn and lacerated character. All of the other organs, both thoracic and abdominal, were normal. And here let me ask, may not many more cases of dysenteric patients than we may suppose, dying suddenly, terminate in this manner?

